# The effect of a digital health coaching and health education protocol on cognition in adults at-risk for Alzheimer’s

**DOI:** 10.1007/s11357-022-00711-3

**Published:** 2022-12-17

**Authors:** Anthony Campitelli, Joshua L. Gills, Megan D. Jones, Sally Paulson, Jennifer Myers, Kelsey Bryk, Erica N. Madero, Jordan M. Glenn, Charlie H. Rodgers, Jenova A. Kempkes, Michelle Gray

**Affiliations:** 1 Department of Health, Human Performance and Recreation, University of Arkansas, Fayetteville, AR USA; 2 St. Elizabeth Healthcare, Edgewood, KY USA; 3 Neurotrack Technologies, Inc, Redwood City, CA USA

**Keywords:** Health coaching, Cognition, Alzheimer’s disease, Alzheimer’s risk

## Abstract

Several modifiable lifestyle factors have been linked to cognitive ability and the risk of developing Alzheimer’s disease and related dementias (ADRD). Health coaching (HC) is an intervention that addresses lifestyle factors associated with cognition. The effectiveness of an HC protocol was evaluated and compared with a health education (HE) intervention, representing the current standard of care, in a sample of 216 adults between the ages of 45 and 75 years who were at-risk for developing ADRD. Outcomes examined were global cognition, neuropsychological cognition, and Alzheimer’s risk. HC participants received personalized coaching from a health coach focusing on nutrition, physical activity, sleep, stress, social engagement, and cognitive activity. HE participants received biweekly education materials focusing on the same modifiable lifestyle factors addressed by HC. Participants were assessed at baseline and again 4 months later. Self-reported global cognition scores improved only in the HC group (16.18 to 15.52, *p* = .03) and neuropsychological cognitive ability improved in the HE group (104.48 to 108.76, *p* < .001). When non-adherence in the HC group was accounted for, however, the mean change in neuropsychological score was similar between groups (*p* > .05), self-reported global cognition demonstrated an even larger mean improvement in the HC group (16.20 to 15.41, *p* = .01), and the HC group saw an improvement in ADRD protective risk score (− 10.39 to − 11.45, *p* = .007). These results indicate that HC and HE can both improve cognition, but HC may be more effective and may yield increased protection against ADRD risk.

## Introduction

Alzheimer’s disease and related dementias (ADRD) represent a major risk to both quality and quantity of life in older adults, as well as imposing a major economic cost both individually and societally [1]. Currently, ADRD affects more than 6 million Americans, and by the year 2050, this number is expected to rise to more than 13 million [1]. One in three seniors will die with an active ADRD diagnosis, and annual deaths directly attributed to those diseases outpace deaths caused by breast and prostate cancer combined [1]. ADRD also incurs a substantial economic cost. It is estimated that in 2021, these cognitive diseases cost Americans $355 billion in healthcare expenditures, and this is projected to rise to $1.1 trillion by 2050. The economic costs extend to labor liabilities as well, and in 2020 more than 11 million unpaid caregivers worked 15.3 billion hours providing support for individuals with ADRD—their time is valued at $257 billion in lost wages [1]. These facts illustrate the monumental negative impact ADRD can have on older adults, and thus researchers have investigated many different strategies to improve or maintain cognitive ability in that population. To date, however, interventions designed to improve cognitive health in at-risk participants have only demonstrated a small effect [2–4].

Presently, Alzheimer’s standard of care has focused mainly on reducing disease symptoms pharmacologically [5] and modifying behavior to stave off disease progression to the extent possible while further reducing cognitive symptoms [3, 4]. Most pharmaceutical Alzheimer’s treatments such as cholinesterase inhibitors (donepezil and rivastigmine) and *N*-methyl-d-aspartate antagonists (memantine) focus on relieving disease symptoms rather than slowing, halting, or reversing its progression [5]. Currently, only one drug is approved for use in the USA for the treatment of Alzheimer’s progression (aducanumab), but the declaration of its effectiveness and subsequent approval by the Food and Drug Administration has been met with criticism from the medical research community [6, 7]. Researchers have also utilized behavioral modifications such as improved diet, exercise, cognitive engagement, brain training, and vascular risk monitoring [2–4]. These attempts at behavior modification have demonstrated efficacy in improving cognitive ability among cognitively impaired or at-risk individuals [2, 3] and were demonstrated in one large study to slow the decline in cognition of at-risk older adults [4]. However, in all cases the efficacy of these interventions has been modest. As it stands currently, doctors utilize pharmaceuticals to reliably reduce symptoms of ADRD and educate their patients regarding behavior and lifestyle modifications that may improve cognition and slow cognitive decline progression as the contemporary standard of care (although the latter outcome is less borne out in the research literature) [5].

Due to the underwhelming results of previous research examining interventions targeting clinical populations with ADRD, the current research landscape has shifted substantially in favor of examining interventions targeting at-risk individuals with no current diagnosis of ADRD [4]. It is believed by many researchers that intervening early in the process of cognitive decline, either before any decline has taken place or when the magnitude of decline is marginal (predementia or mild cognitive impairment), may allow for early-stage preventive decrement of ADRD risk and perhaps a reduction in the rate of progression [8, 9]. The examination of which variables, modifiable through intervention, should be addressed in ongoing research examining ADRD mitigation is now the subject of much investigation [9].

Several modifiable lifestyle domains have been identified which elevate risk for ADRD including overweight/obesity, physical inactivity, stress, low cognitive activity, low social engagement, chronic inflammation, poor dietary habits, poor sleep, high blood glucose levels, and high blood lipid levels [10]. Researchers have examined interventions to target improvements in these lifestyle domains as a way to mitigate ADRD risk with varying degrees of success [2–4, 11, 12]. Health coaching (HC) is a guided intervention wherein a health coach assists participants in achieving their desired health-related outcomes through goal-setting, education, motivation, re-assessment, and personalized, guided progression [13]. In previous studies, HC improved many of the lifestyle factors associated with ADRD [14, 15] and may facilitate increased engagement in behaviors believed to benefit cognition [15]. However, HC’s efficacy for specifically decreasing ADRD risk and improving cognitive-related function among an at-risk sample has not, to our knowledge, yet been examined.

With the positive impact of HC being demonstrated in so many areas related to ADRD and the, at most, modest effectiveness of the current ADRD standard of care, it prompts the research question: Will an HC program be beneficial for reducing ADRD risk or improving cognition in at-risk individuals? Also, a secondary question arises: Is there a way to present standard of care in a novel format with measurable effectiveness in that same population? The purpose of this study was to investigate the effect of a 4-month HC intervention on the risk for ADRD and cognitive ability in at-risk individuals. In addition, the educational aspect of standard of care was presented in a remote, digital health education (HE) format to gauge its effectiveness concurrently in a second sample from the same population. The change in cognitive outcomes was also compared between the HC and HE group to determine if one treatment was more efficacious than the other. The research null hypotheses tested were as follows: In adults at-risk for ADRD, (1) the cognitive and Alzheimer’s disease risk scores were not different between baseline and 4-month time points for the HC group, (2) the cognitive and Alzheimer’s disease risk scores were not different between baseline and 4-month time points for the HE group, and (3) there was no difference in overall cognition score change or Alzheimer’s disease risk change between the HC and HE groups. This study is an analysis of preliminary data from the Digital Cognitive Multidomain Alzheimer’s Risk Velocity (DC MARVel) study [16].

## Methods

### Sample

**Fig. 1:**
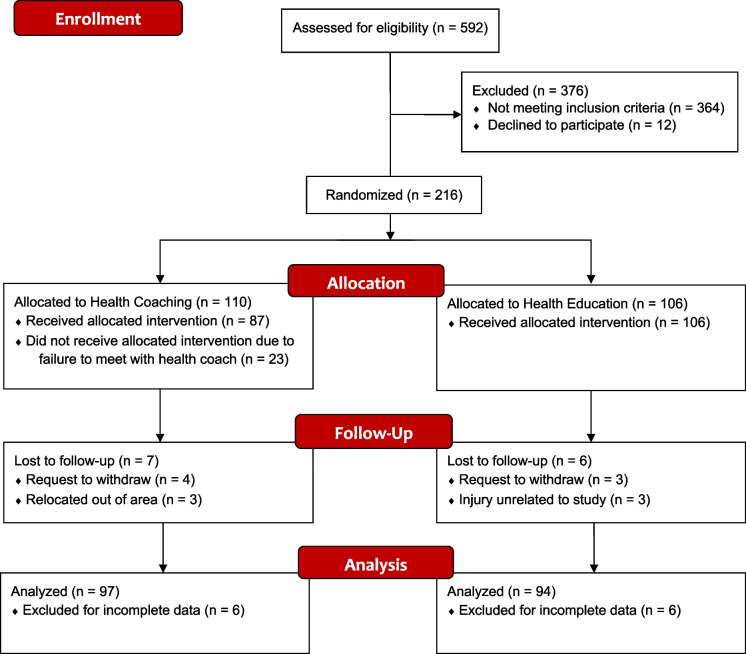
Study recruitment flowchart

A total of 216 adults were recruited for this study out of 592 screened individuals. Due to attrition and missing data, 191 adults were included in the final analyses (138 females and 53 males). A full accounting of recruitment, sample size, and attrition is presented in Fig. 1. To be included, a participant had to be between the ages of 45 and 75 years, be fluent in English, own a smartphone, be willing to communicate via text message, and have at least two of the following risk factors for ADRD based on the Australian National University–Alzheimer’s dementia risk index (ANU-ADRI): high school education or less; a body mass index (BMI) ≥ 25 kg/m^2^ but less than 40 kg/m^2^; or history of diabetes, hypertension, high cholesterol, smoking, or traumatic brain injury. Participant exclusion criteria were visual problems impacting the ability to view a screen at a normal distance; history of a learning disability; recent cardiovascular event; current participation in a cognitive training intervention or lifestyle change program; current diagnosis of any mental health condition, neurologic condition, dementia, mild cognitive impairment, or any other serious health condition; or more than one of the following ADRD protective factors based on the ANU-ADRI: high physical activity level, eating non-fried fish or seafood more than 5 times per week, or a high level of cognitive engagement.

Required sample size was determined a priori by utilizing both a mathematical and practical approach. Mathematically, total sample size requirement was calculated using G*Power 3.1.9.7 [17] based on the primary statistical test planned for this study (2 × 2 mixed factorial ANOVA). Calculations were based on an alpha level of 0.05, a statical power of 0.8, and a small effect size (*η*_*p*_^2^ = 0.02 or *d* = 0.2). The largest required sample size returned from mathematical analyses was a total sample of 100 total participants. Practical analyses for required sample size were conducted by examining related HC literature and the samples recruited to demonstrate efficacy therein. Studies examining the efficacy of HC on improving psychological and cognitive variables were conducted with sample sizes ranging from 40 to 45 total participants and demonstrated significant improvement in outcome measures [14, 15, 18]. Taken together, a sample size of at least 100 participants was pursued for this study.

A convenience sampling procedure was utilized in this study. Participants were recruited through advertising on National Public Radio, advertising on a university newswire service, social media, and word of mouth. Potential participants expressing interest in the study were emailed a link to an inclusion/exclusion survey instrument which was used to determine whether they were a candidate for the study.

### Study design

This study followed a parallel arm trial design wherein participants were randomized into one of two groups (HC or HE) with an equal allocation ratio and then scheduled for an initial visit to the laboratory to complete a testing session. Randomization was achieved by pre-assigning all study IDs to an arm using a binary random number generator with an equal allocation randomization rule for the full recruited sample size. As participants entered the study, they were sequentially assigned an ID number and assigned to the corresponding study arm. No form of blinding was utilized in this trial. The randomization sequence was generated by the principal investigator, and study enrollment as well as formal trial arm assignment was carried out by the study coordinator.

### Measures

Three primary outcomes were assessed in this study. First, the participant’s self-reported global cognition was collected as a measure of their own perception of their cognitive state. Self-reported global cognition, although subjective, is a valid and reliable predictor of cognitive state [19] and is sensitive to detection of mild cognitive impairment [20]. Second, neuropsychological cognition was assessed as an overall objective measure of a participant’s cognitive state. Multidimensional neuropsychological cognition assessment batteries are the accepted gold standard for objective cognitive assessment, and these instruments are generally used in both the research and clinical setting to discriminate between individuals with and without cognitive impairment [21, 22]. Third, Alzheimer’s risk was assessed as both a risk measure (positive risk), protection measure (negative risk), as well as a composite risk score taking into account both risk and protection. In long-term follow-up studies, Alzheimer’s risk data obtained from survey instruments has good predictive validity for determining future ADRD diagnoses within 3–6 years following testing [23].

#### Global cognition

The Everyday Cognition (ECog-12) survey was used to assess self-reported global cognition. The ECog-12 is a self-reported survey instrument that asks participants to compare their current state of cognition to their cognitive state 10 years in the past [19]. Each of the survey’s 12 items use a 4-point scale, with higher values indicating greater cognitive impairment [19]. The ECog-12 is reliable and valid, and has excellent discriminant ability for separating participants with clinical cognitive impairment from individuals with normal cognitive function [19].

#### Neuropsychological cognitive ability

The Repeatable Battery for the Assessment of Neuropsychological Status (RBANS) was used to assess neuropsychological cognition. The RBANS assessment is a digital assessment presented to participants on a tablet and administered by a trained test administrator. Participants are asked to complete several cognitive/memory tests including repeating words and stories, drawing geometric shapes, identifying pictures, and matching symbols with numbers from a given key [24]. The assessment evaluates five neuropsychological construct domains: immediate memory, visuospatial/constructional, language, attention, and delayed memory. Scores from the five domains were adjusted based on age and level of education, and combined to yield a single, continuous index score indicating overall neuropsychological cognitive ability [24]. RBANS is a valid and reliable instrument for measuring neuropsychological cognitive domains and overall ability [25].

#### Alzheimer’s risk

The ANU-ADRI is a self-report inventory assessing Alzheimer’s risk across several positive and negative risk factors [10]. Protective (negative risk) factors examined are social engagement, cognitive activity, physical activity level, non-fried fish and seafood consumption, and alcohol consumption (if less than 2 drinks per day). Risk (positive risk) factors assessed in this survey are diabetes diagnosis, depression status, obesity, history of traumatic brain injury, history of smoking, high cholesterol, high alcohol consumption (3 or more drinks per day), exposure to pesticides, as well as known demographic risk factors such as sex, age, and level of education [10]. The ANU-ADRI is a valid [23] and reliable [26] measure of Alzheimer’s risk.

### Data collection

Participants who were identified as candidates for inclusion in the study upon completion of the initial inclusion/exclusion survey were asked to complete a digital copy of an informed consent document. The study, all recruiting procedures, and informed consent were approved by the Institutional Review Board at a major, land-grant institution of higher education. Participants returned a digitally signed informed consent. In addition to this first session, participants completed a second session approximately 4 months from the first.

Prior to arriving for each testing visit, participants were asked to complete one additional digital survey remotely to collect demographic and health status data. This demographic and health survey contained the ECog-12 survey and questions designed to collect relevant demographic information such as age, sex, and level of education. Other information was collected by the demographic and health survey which was not utilized in the present analysis including healthcare utilization, diagnosed medical conditions, prescribed medications, self-reported health status, depression status, and sleep quality status. If this survey was not completed remotely before each appointment, completing it was the first task assigned to participants upon arrival at the laboratory.

Arriving at the laboratory (or after completing the demographic and health survey if not completed beforehand), participants were asked to complete the ANU-ADRI assessment on a provided laptop. Although the inventory is self-guided, a researcher was present to answer any questions the participant may have had regarding the ANU-ADRI. Following completion of the ANU-ADRI, basic cardiovascular and anthropometric data were collected from participants. Blood pressure was collected manually by a trained researcher using a standard inflatable sphygmomanometer cuff and stethoscope, and pulse rate was collected with a standard pulse oximeter on the finger. Weight was collected using a beam-balance physician’s scale, and height was collected using a stadiometer.

The RBANS assessment was administered roughly 20 min after the cardiovascular and anthropometric assessments. The RBANS test is produced in multiple versions utilizing the same battery of tests but make slight changes to the presentation of words, figures, and numbers to mitigate learning effects from test to re-test [24]. For this study, the RBANS Form A was administered in the first appointment and Form B in the second. The RBANS tests were graded by a trained and experienced rater in accordance with procedures from the RBANS manual [24]. Data were recorded by researchers and input into a master database for analysis.

As this present analysis was performed as a part of a larger study, other data were collected but not utilized here. Participants completed other measures of body composition and physical function that are not germane to the analysis presented here. The full protocol for the DC MARVel Study is described elsewhere [16].

### Intervention

#### Health coaching

Participants randomized into the HC intervention were assigned to a trained health coach who worked with them throughout the study’s duration. HC is unique in the set of health and lifestyle interventions typically examined in research in that it does not follow a standardized approach. Rather, it works within a set of principles and practices to provide a personalized intervention to participants. After the first visit to the laboratory, participants were scheduled to have an initial video conference or phone call with their health coach wherein they discussed the HC process, were educated about lifestyle domains and their impact on cognitive health, described to the coach which domains they wanted to change, assessed their motivation and willingness to change, and established goals to achieve their desired future vision. The health coach focused on improving cognitive health through targeting the following lifestyle domains: nutrition, physical activity, sleep, stress, social engagement, and cognitive activity. The specific intervention for each participant is formulated within that framework and the decision of which modifiable risk factors to focus on is made based on the participant’s preferences and the coach’s recommendation.

During the intervention, the participant and coach communicated monthly via video conference or phone call, and the coach reached out to participants 1–2 times per week via text messaging app and email. In monthly meetings, the coach checked progress, assessed readiness for progression, discussed obstacles, and strategized about how the intervention would be implemented subsequently. More frequent weekly messages and emails to participants would provide personalized education materials based on the specific participant’s current goals. HC participants were also provided access to a cognitive health app (Citruslabs, Santa Monica, CA), where they can access cognitive training activities, workout routines, and recipes, and were instructed to interact with the app at least 3 times per week. Adherence to the HC protocol was defined as the completion of at least one HC appointment between baseline and the 4-month time point. The health coach recorded all adherence data for each de-identified participant in an online database.

#### Health education

Participants randomized into the HE intervention received a biweekly email that included educational material outlining how they could improve cognitive health through lifestyle change. Participants were asked to read each email when they received it, and the emails were designed to be eye-catching and engaging. The same lifestyle domains addressed in HC were utilized as topics in HE to allow for better direct comparison between interventions without adding an additional source of variability. Outside of scheduling and basic communication, HE participants only had access to study staff during their on-site testing appointments.

### Data analysis

All data analyses were performed using SPSS 27 (IBM Corp, Armonk, NY). Means, standard deviations, and 95% confidence interval of the mean were calculated for all continuous dependent, demographic, and anthropometric variables. Before completing any inferential statistical tests, all relevant assumptions were checked, and if they were met, statistical analysis was allowed to proceed. Prior to hypothesis testing, an independent samples *t*-test was utilized to determine if there were differences in baseline cognitive scores (ECog-12 and RBANS) due to sex. Another independent samples *t*-test determined if cognitive score differences existed between the HC and HE group at baseline. Dependent samples *t*-tests were utilized to determine if outcomes had changed from baseline to 4 months with each intervention group (HC and HE). A 2 × 2 (intervention × time) mixed factorial ANOVA was utilized to determine if a difference in cognitive scores obtained in the first and second visit was dependent on intervention (HC or HE). That is, to determine if there was a significant intervention × time interaction effect. If baseline sex differences were found in cognitive scores, sex was included as a blocking factor to better isolate the effect of intervention and time. An a priori alpha level of 0.05 was used for all analyses.

## Results

Demographic statistics for the overall sample are presented in Table 1, inclusive only of participants who had complete data for the outcome variables. Descriptive statistics from baseline and time 2 are presented in Table 2. An independent samples *t-*test revealed that baseline sex differences were present for RBANS total score (*t*_*ADJ*_(81.24) = 2.82, *p* = 0.006). In the test for baseline sex differences, the assumption of homogeneity of variance was violated for this test (folded *F*(58,154) = 1.99, *p* = 0.001) and a Satterthwaite correction was utilized. Sex, therefore, was utilized as a blocking factor in further inferential tests examining differences in RBANS total scores to partial out its influence from the model. No baseline sex differences were found for ECog-12 global cognition score (*t*(216) = 0.86, *p* = 0.39). Likewise, ANU-ADRI total score demonstrated no sex differences at baseline (*t*(213) = 0.59, *p* = 0.55), nor did its component risk (*t*(213) = 0.45, *p* = 0.66) and protection (*t*(213) = 0.41, *p* = 0.69) scores. No differences between intervention group means were found at baseline for any dependent variable (*p* > 0.05). Correlations of the age of participants in this sample with any of the outcome measures examined in this study were all small (|*r*|< 0.19).

**Table 1:** Demographic descriptive statistics (*n* = 191)

Variable	Mean	SD	95% Confidence interval	
			Lower bound	Upper bound
Age (years)	61.94	8.23	60.84	63.05
Height (cm)	167.50	9.13	166.27	168.72
Mass (kg)	84.89	18.23	82.45	87.34

**Table 2:** Time 1 and 2 descriptive statistics (*n* = 191)

Variable	Mean	SD	95% Confidence interval			
			Lower bound		Upper bound	
RBANS total score (T1)	104.61	13.56		102.62		106.60
RBANS total score (T2)	107.83	12.72		105.96		109.69
ECog-12 score (T1)	16.48	4.11		15.87		17.08
ECog-12 score (T2)	16.14	3.99		15.55		16.72
ANU-ADRI total (T1)	− 1.77	7.39		− 2.85		− 0.68
ANU-ADRI total (T2)	− 1.97	7.57		− 3.08		− 0.86
ANU-ADRI risk (T1)	8.73	5.82		7.88		9.58
ANU-ADRI risk (T2)	8.82	5.95		7.94		9.69
ANU-ADRI protective (T1)	− 10.50	4.37		− 11.14		− 9.86
ANU-ADRI protective (T2)	− 10.79	4.41		− 11.44		− 10.14

Scores for each instrument are presented in pairs. Timepoint is indicated for baseline (T1) and 4 months (T2). No significant group-pooled main effects for time were observed for any dependent variable (*p* > .05)

**Fig. 2:**
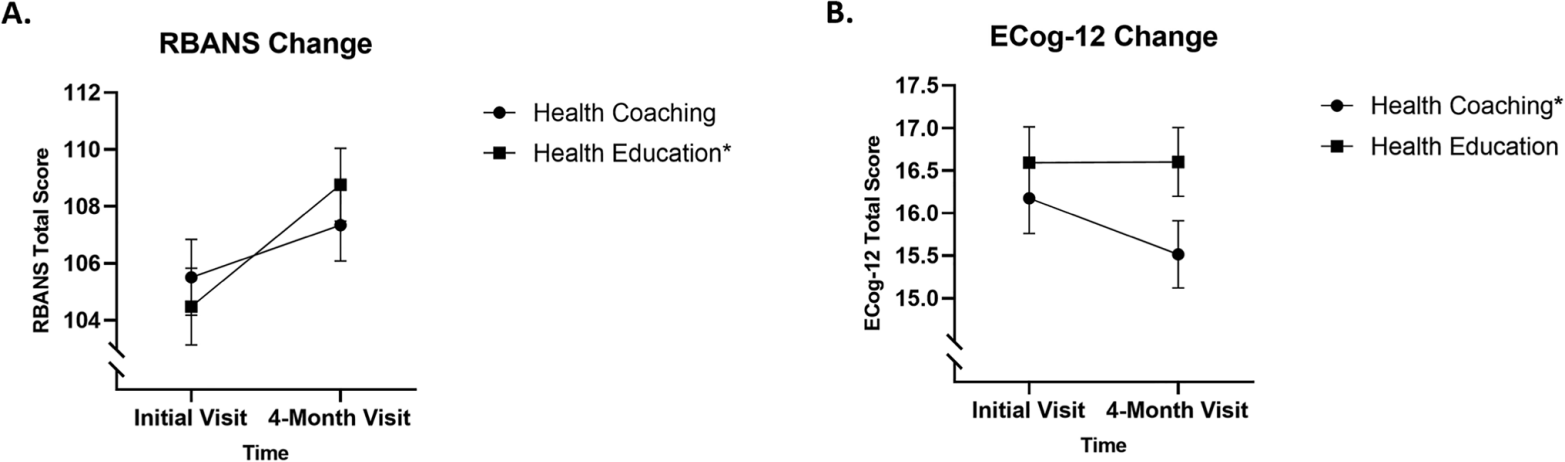
Change in RBANS total score and ECog-12 score for each group from baseline to 4-month follow-up. **A** The change in RBANS total score from baseline to time 2 for each group. **B** The change in ECog-12 total score from baseline to time 2 for each group. The axes have been broken to better show change. Error bars indicate standard error. Asterisk (*) indicates that a group changed significantly from baseline to time 2 (*p* < .05)

Assumptions were checked prior to performing all inferential statistical tests. Normality was checked for all treatment × time cell datasets utilizing a Shapiro–Wilk test. The distribution of data in several cells were found to be significantly different from a normal distribution (*p* < 0.05). It was determined, however, that data would not be transformed to address the departure from normality as cell skewness and kurtosis values were not extreme, the assumption of homogeneity of variance was met in all cases, and ANOVA is robust to violation of normality in this context [27]. Homogeneity of variance was checked for each dependent variable using a Brown–Forsythe test as there were non-normal cell distributions of data [27], and in all cases the assumption of homogeneity of variance was met (*p* > 0.05). The assumption of independence was checked with a Durbin–Watson test for each dependent variable, and in all cases the assumption of independence was met (*D* = 1.65 to 1.74). ANOVA results revealed that RBANS total score did not change from baseline to time 2 when HC and HE were pooled (*F*(1,194) = 0.31, *p* = 0.58, *η*_*p*_^2^ = 0.002). However, there was a significant treatment × time interaction effect (*F*(1,194) = 3.99, *p* = 0.047, *η*_*p*_^2^ = 0.020) indicating that treatment groups did change at a different rate from baseline to time 2. Pairwise comparisons revealed that *only* the HE group improved in RBANS total score from baseline to time 2 (104.48 to 108.76, *p* < 0.001). Overall, ECog-12 global cognition score did not change significantly from baseline to time 2 (*F*(1,188) = 2.57, *p* = 0.11, *η*_*p*_^2^ = 0.013). Although a statistically significant treatment × time interaction effect was absent, it is worth reporting that a dependent samples *t*-test revealed that only the HC group improved in ECog-12 score (16.18 to 15.52, *t*(96) = 2.23, *p* = 0.03). ANU-ADRI total score (*F*(1,198) = 0.24, *p* = 0.63, *η*_*p*_^2^ = 0.001), risk score (*F*(1,198) = 0.24, *p* = 0.63, *η*_*p*_^2^ = 0.001), and protective score (*F*(1,198) = 1.16, *p* = 0.282, *η*_*p*_^2^ = 0.006) did not experience a statistically significant change from baseline to time 2. As an intra-group time main effect was found in the variables, the change in RBANS total score and ECog-12 score for each group from baseline to 4-month follow-up are presented graphically in Fig. 2.

Adherence appeared to be poor in a large segment of the HC group. Upon examination, it was discovered that many participants (*n* = 23) had not completed a single scheduled HC visit with the health coach between time points. These cases were defined as non-adherents, removed from the data set, and a set of secondary analyses were conducted to gauge how only examining HC-adherent participants might change the outcome of the analyses.

**Fig. 3:**
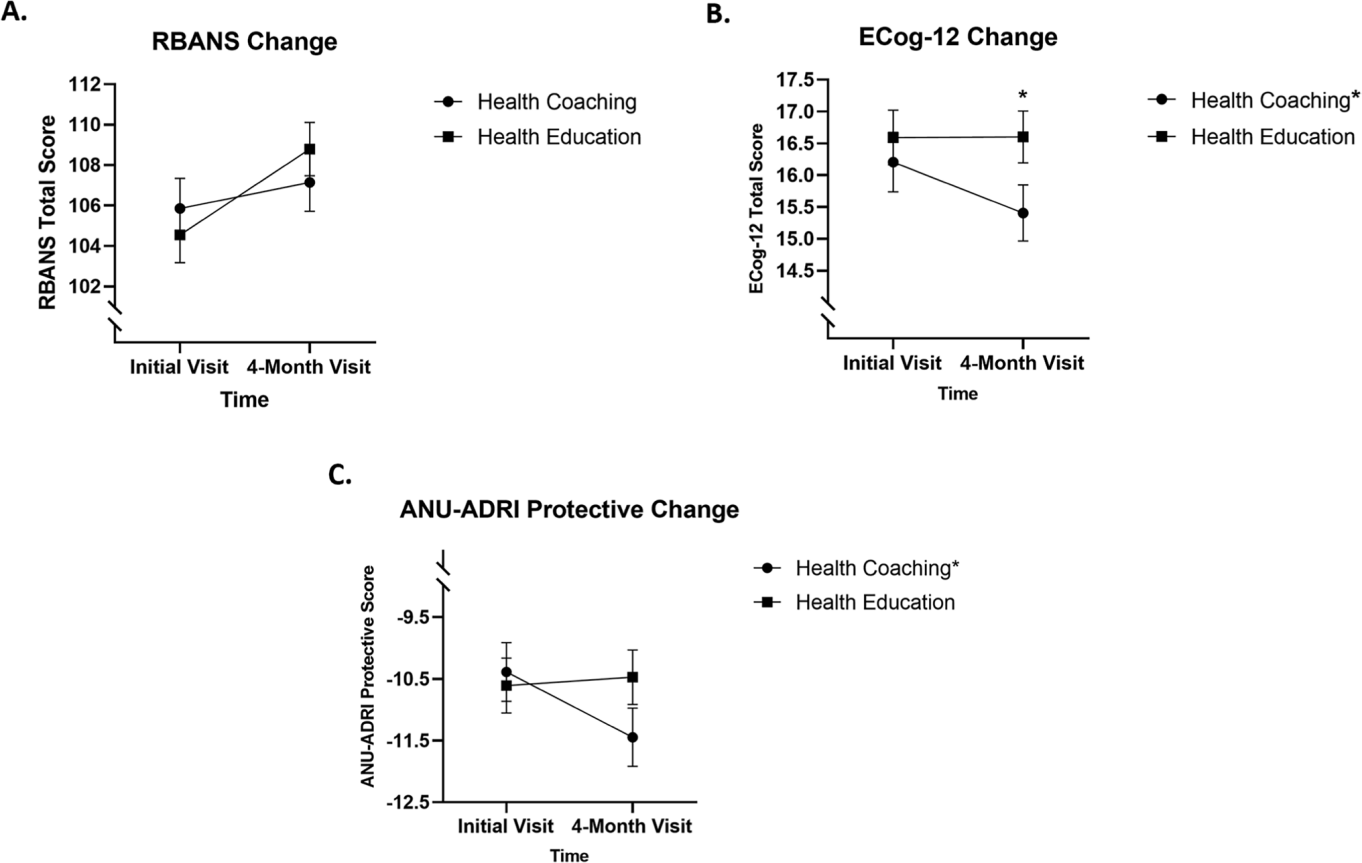
Treatment group changes from baseline to time 2 in RBANS total score, ECog-12 score, and ANU-ADRI protective score for the adherent-only data set. All figures for analyses not including non-adherent HC participants. **A** The change in RBANS total score from baseline to time 2 for each group. **B** The change in ECog-12 total score from baseline to time 2 for each group. **C** The change in ANU-ADRI protective score from baseline to time 2 for each group. The axes have been broken to better show change. Error bars indicate standard error. Asterisk (*) next to a group name indicates that a group changed significantly from baseline to time 2 (*p* < .05), and asterisk above a figure marker indicates a significant difference between groups at that time point (*p* < .05)

After removal of the non-adherent participants, statistical analyses were conducted again. When non-adherent HC participants were removed from the analysis, the previously observed treatment × time interaction effect for RBANS total score was no longer statistically significant (*F*(1,177) = 3.25, *p* = 0.07, *η*_*p*_^2^ = 0.017), indicating that any change from baseline to time 2 was not dependent on treatment group. Dependent samples *t*-tests indicated that RBANS total scores improved for both HE and HC in the adherent-only group from baseline to time 2 (*p* < 0.05). The effects on ECog-12 score were magnified by removing non-adherent participants. In this re-analysis, a significant treatment × time interaction effect was observed in ECog-12 score (*F*(1,170) = 3.98, *p* = 0.048, *η*_*p*_^2^ = 0.022), and pairwise comparisons revealed that improved ECog-12 scores from baseline to time 2 were only observed in the HC treatment group (16.20 to 15.41, *p* = 0.010). Also, the ECog-12 score for the HC group was significantly better than the HE treatment group at the 4-month time point (*p* = 0.048) whereas this difference was not present in the full data set. The removal of non-adherents also affected the analysis of ANU-ADRI protective scores. In the adherent-only re-analysis, a significant treatment × time interaction effect was observed for ANU-ADRI protective score (*F*(1,180) = 5.02, *p* = 0.03, *η*_*p*_^*2*^ = 0.027) indicating that change in protective score from baseline to time 2 was dependent on treatment group. Pairwise comparisons revealed that protective score only improved in the HC group from baseline to time 2 (− 10.39 to − 11.45, *p* = 0.007). Treatment group changes from baseline to time 2 in RBANS total score, ECog-12 score, and ANU-ADRI protective score for the adherent-only data set are presented graphically in Fig. 3.

No adverse events were reported during this trial for either study arm.

## Discussion

In the overall dataset, scores in the HE group improved over the 4-month study but remained the same in the HC group. The mean ECog-12 score for the HC group improved over the 4 months while the HE group’s mean score did not change for that variable. A significant treatment × time interaction effect was detected for RBANS total score, indicating that it improved significantly more in the HE group from baseline to the 4-month visit compared to the HC group. No other time differences, group differences, or interaction effects were observed in the overall dataset. Results from the full dataset indicated that a remote, digital HE protocol was effective in improving neuropsychological cognition, while HC improved self-reported global cognitive ability.

Based solely on inferences derived from the full dataset, it appeared that both HE and HC had a positive effect on different aspects of cognition in this study’s sample. Telling the story of these findings, however, hinges greatly upon the impact of adherence in the HC treatment group. When individuals who were not adherent to the HC protocol (participants who did not complete a single HC visit in the 4-month study) were removed from the study, the inferences changed relatively extensively. First, the advantage of HE in improving RBANS score (significant treatment × time interaction effect) was no longer observed and both groups showed improved RBANS scores over the 4 months of study. Differences in ECog-12 score became even more pronounced when non-adherent participants were removed from the analysis with HC improving significantly more than HE. Finally, ANU-ADRI protective score improved only in the HC group when only adherent participants were analyzed. To summarize, when looking at only individuals who adhered to the HC protocol by completing at least one HC session, HC appeared to be superior to HE in improving self-reported global cognition, increasing modifiable ADRD-protective behaviors, and equally effective improving neuropsychological cognitive ability.

Generally, effect sizes observed were small but measurable (*η*_*p*_^2^ = 0.020 to 0.028). These small effect magnitudes are generally not out of the ordinary for research investigating changes in cognition in response to cognitive engagement interventions [2–4] and are in-line with specific effect sizes from previous literature [28]. In addition, these results, although meaningful, are preliminary. As such, there is the potential that the participants in this study may still be adapting to the intervention and may show further improvements as the study proceeds.

It seems that HC is an effective way to improve cognition and may be superior to traditional HE interventions. These results support previous research that speculated HC may have the potential to improve cognitive ability [15]. This study builds upon prior findings by demonstrating that the behavior change realized through HC interventions may be accompanied by measurable improvements in cognition in a population at-risk for ADRD. Furthermore, these findings indicate that protection against Alzheimer’s risk (negative risk factors) are improved by a HC intervention as well. This is a particularly interesting phenomenon due to the nature of the instrument used to measure risk. The ANU-ADRI was validated as a way to approximate latent Alzheimer’s risk which cannot be measured directly [23]. This is of particular interest here, but the protective score component of the ANU-ADRI is also ultimately a composite of modifiable behaviors which have shown to be protective against Alzheimer’s risk. This single result suggests that HC is simultaneously effective for hedging against risk and initiating real behavior change in adhering individuals. This result supports those of previous studies demonstrating that HC increases performance of positive health behaviors associated with cognitive health [14, 15].

Regarding HC, the importance of adherence should not be overlooked. The dramatic change in the statistical inferences derived from this study after controlling for complete non-adherence (even without further parsing for level of adherence beyond its absence) is a testament to the potential importance of adherence in HC. HC literature, unfortunately, often does not account for adherence to the protocol, but the impact of adherence observed in this study supports similar observations made in other research [29, 30]. Even outside of this specific context of ADRD and cognition, adherence may be an important factor in determining overall HC treatment effectiveness. Future studies examining HC interventions ought to consider the role of adherence in the outcome, and strategies to maximize adherence will likely improve a planned HC paradigm.

It is also worth noting that the HE protocol used in this study was different from a traditional HE model in key ways: it offered a repeated, consistent engagement schedule with participants, and it allowed for completely remote, large-scale, digital dissemination. These features lend a novel element to the HE system utilized here, making it worthy of evaluation. Here, the finding that HE was efficacious in improving overall neuropsychological cognition score in this sample should be highlighted. While HC, at this juncture, appears to be generally more positively impactful on participants’ cognition and fortifying against ADRD risk, HE’s effectiveness opens up a potential treatment option for patients who lack the resources to afford HC or lack access to a HC program.

A potential limitation of this study is the sampling method utilized. Although the sample included many unique individuals, the sampling method was not random and, as a result, the resulting sample may not be representative. The 2.6:1 observed ratio of females to males in this sample is evidence of a non-representative sample. Although the results of this study are promising for cognitive health interventions, caution should be exercised regarding the broad application and generalization of these results. The requirement that participants be fluent in English and use a smart phone may be a source of sampling error and reduced generalizability to all populations as previous research has shown the acquisition of vocabulary impacts cognition [31] and that memory and attention can be affected by smartphone usage [32]. Although non-adherents were removed, poor adherence was another potential limitation as it prevented the inclusion of the full sample in the second set of analyses. Future research performed by the authors will focus on examining the effectiveness of a HC intervention over a longer time horizon (2 years vs. 4 months). Improved adherence will be prioritized as well, and the impact of HC and HE on other variables such as ADRD biomarkers and physical function will be examined.

These preliminary results demonstrate HC’s potential as a valuable and comprehensive ADRD intervention for at-risk individuals. While participants who engaged in the HC intervention (adherence) had better outcomes than those in in the HE group, findings showed a structured HE paradigm may also provide some cognitive benefit. As the cost of ADRD continues to rise, preventative interventions such as HC may serve as the best chance of reducing or delaying ADRD.
